# Rational Polytherapy with Antiepileptic Drugs

**DOI:** 10.3390/ph3082362

**Published:** 2010-07-26

**Authors:** Jong Woo Lee, Barbara Dworetzky

**Affiliations:** The Edward B. Bromfield Epilepsy Program, Division of EEG, Epilepsy, and Sleep Neurology, Department of Neurology, Brigham and Women’s Hospital, Boston, MA 02115, USA; E-Mail: bdworetzky@partners.org (B.D.)

**Keywords:** antiepileptic drug, rational polytherapy, epilepsy

## Abstract

Approximately 30–40% of patients do not achieve seizure control with a single antiepileptic drug (AED). With the advent of multiple AEDs in the past 15 years, rational polytherapy, the goal of finding combinations of AEDs that have favorable characteristics, has become of greater importance. We review the theoretical considerations based on AED mechanism of action, animal models, human studies in this field, and the challenges in finding such optimal combinations. Several case scenarios are presented, illustrating examples of rational polytherapy.

## 1. Introduction

Until the early 1980’s, polytherapy was widely practiced as first-line treatment for refractory epilepsy with the hope of achieving synergistic effects or less severe drug toxicity on smaller doses of two drugs rather than larger doses of one drug [1]. Subsequent trials led to a change in this method by validating monotherapy as first-line treatment [2]. Despite advances in the treatment of epilepsy, 30–40% of patients remain uncontrolled on a single anti-epileptic drug (AED) [3]. For these patients, polytherapy is not only acceptable, but is standard practice [4]. Prior to 1993, there were only a few AEDs available for the treatment of partial epilepsy. These included valproic acid (VPA), carbamazepine (CBZ), phenytoin (PHT), phenobarbital (PB), and benzodiazepines (BZD). Therefore, there were only 10 possible two-drug combinations, a daunting but not impossible combinatorial task, especially if one or two medications could be eliminated from the possibilities. Since 1993, more than 10 additional AEDs have come into existence. If the decision to choose an optimal first-line AED was difficult, finding an optimal two (or more) drug combinations, even by brute force, is challenging. Rational polytherapy, the process of selecting drug combinations with results superior to selecting drugs at random, may remain an art, but quantifiable strategies for arriving at the most effective possible combination are clearly needed. The potential advantages of rational polytherapy are to achieve better seizure control with fewer side effects, as well as control of multiple seizure types that respond to different drugs [5].

## 2. Inherent Difficulties of Polytherapy

The choice of optimal polytherapy poses difficulty for several reasons. First and foremost, there are limited data regarding favorable or unfavorable combinations. With few exceptions, there is little systematic evidence that any two combinations are any more or less effective as any other. 

The clinician’s choice of polytherapy is, in part, determined by his/her comfort with the AED chosen first for monotherapy. This may result in combinations of AEDs that are idiosyncratic for each practitioner, and not necessarily optimally selected for the patient requiring polytherapy. Patients may end up on the specific polytherapy because of the order of introduction of the AED to the market or, in some countries, by funding mechanisms or government subsidies. Although few practitioners would currently choose the combination of PB and PHT as first-line polytherapy for epilepsy, many patients are currently taking these medications in combination as evidenced by retrospective studies [7,8]. Switching off these AEDs if seizures are controlled can be more difficult, sometimes not advisable, as there is a risk of seizure recurrence. The possible gains in bone density over a lifetime from withdrawing from these inducers of the P450 system may ultimately prove to be substantial and worth the effort, but until then these choices are difficult.

Polytherapy may also be governed by the relative ease or difficulty of establishing a specific combination of drugs. For example, the addition of VPA to lamotrigine (LTG) is a challenging tasks for physicians because of the pharmacokinetic interactions necessitating careful reduction in the LTG dose. In comparison, the addition of levetiracetam (LEV) or gabapentin (GBP) to LTG is simple due to the lack of any drug-drug interactions. Even when the desired combination of efficacy and side effect profile is suboptimal, ease of administration may dictate the individual components of polytherapy. 

Lastly, medication side effects are less thoroughly assessed than effectiveness of medications during the patient follow-up visit [6,7]. When patients fail two or more trials of monotherapy and the physician prepares to combine medications, efficacy of any AED will be of even greater importance than medication side effects, even though AED polytherapy has been found to be the strongest indicator for patients experiencing subjective side effects in some studies [6]. Once an effective combination is found, it becomes difficult to reduce medication to act upon side effect complaints, even if they are persistent and debilitating or are known to cause insidious chronic side effects such as osteoporosis [8]. Patients and their doctors do not always agree on the need for these adjustments, especially when AED reduction leads to physician advice to refrain from driving due to concern for possible seizure recurrence. Furthermore, it is quite possible, even likely, that more optimal AED combinations that are both effective and tolerable may not be found.

## 3. Theoretical Considerations

The mechanisms of action of the major AEDs are summarized in Table 1. The concept of choosing polytherapy based on the individual drug’s mechanism of action may, in practice, be difficult to implement, since many AEDs have multiple mechanisms. However, most of the AEDs have a primary mechanism of action, and much attention has been attributed to this characteristic. An unresolved issue is whether AEDs with different mechanisms of action are more likely to interact synergistically than AEDs with similar or differing mechanisms [11].

**Table 1:** AED mechanisms [9,10].

Na channel	**CBZ**
	**FBM**
	**LAC**
	**LTG**
	**OXC**
	**PHT**
	**RUF**
	**TPM**
	**VPA**
	**ZNS**
Ca channel	**ESX** (low voltage T-type)
	**GBP** (high voltage)
	LTG (high voltage N-type)
	OXC
	**PGB** (high voltage)
	PB
	PHT
	**TPM** (high voltage L-type)
	**VPA** (low voltage T-type)
	**ZNS** (high voltage T-type)
GABA enhancers	**Benzodiazepines**
	**PB**
	CBZ
	GBP
	PGB
	**TGB** (GABA uptake inhibitor)
	TPM
	**VIG** (GABA transaminase inhibitor)
	**VPA**
Glutamate antagonists	CBZ
	FBM
	OXC
	PB
	TPM
Carbonic anhydrase inhibitors	TPM
	ZNS
Other	CBZ (adenosine receptor binding)
	LAC (collapsin responsive mediator protein-2)
	**LEV** (synaptic protein SV2A binding)
	PHT (inhibit neurotransmitter release)

Abbreviations: CBZ: carbamazepine; ESX: ethosuximide; FBM: felbamate; GBP: gabapentin; LEV: levetiracetam; LTG: lamotrigine; OXC: oxcarbazepine; PB: Phenobarbital; PHT: phenytoin; RUF: rufinamide; TGB: tiagabine; TPM: topiramate; VIG: vigabatrin; VPA: valproic acid.

Combination therapy with complementary mechanisms of action has generally been recommended [4]. There is the theoretical consideration that small effects on multiple drug targets may be more optimal than targeting a single mechanism of action [12]. Studies have shown that the combination of PHT and CBZ, both sodium channel blockers, is less effective than with either in combination with PB [13]. However, in a meta-analysis of 536 animal studies, Jonker *et al*. [14] did not find any combination of mechanisms more or less likely to be synergistic, with the exception of the inclusion of an AMPA-receptor antagonist, few of which are currently marketed. To complicate matters, other studies have postulated that AEDs with similar mechanisms of action are more effective than two AEDs with different mechanisms [15]. Synergy between two GABAergic drugs, tiagabine and vigabatrin, has been demonstrated in brain slice experiments [16] as well as in a limited number of patients [17]. Although structurally and mechanistically similar, oxcarbazepine (OXC) added to CBZ has also been shown to be effective [18]. Of note, AEDs presumed to have similar pharmacology (e.g., pregabalin and GBP) have perhaps been unnecessarily eliminated from testing in some adjunctive randomized clinical trials [19,20]. 

It has been postulated that selection of AED combinations by mechanism of action may be useful because AEDs with similar mechanisms may have similar side effect profiles. Such AEDs in combination may cause an excessive amount of additive side effects when used in combination. For example, the use of CBZ and OXC may exacerbate hyponatremia, or two BZDs may cause excessive drowsiness [11]. In fact, there is greater evidence of pharmacodynamic interaction for the side effect profile than for efficacy [21]. However, the mechanisms of action responsible for the antiepileptic effects may not be the same ones that are responsible for the side effects. Some studies have shown that even drugs that are thought to have similar mechanisms of action such as PHT and CBZ manifest different neurotoxic profiles [22]. Furthermore, even if two drugs with completely different mechanisms of antiepileptic action are selected, they may each be prone to similar side effects and in combination cause exaggerated additive/supra-additive side effects by acting upon common but unidentified targets unrelated to their antiepileptic mechanisms [23]. The fact that some generic formulations cause side effects not seen in the brand name medication argues that even non-active ingredients may contribute to side effects [24].

In summary, mechanisms of action have not yet provided meaningful guidelines to aid in the rational choice for polytherapy as there is insufficient evidence to determine whether identical or complementary mechanisms should be targeted. Unfortunately, this appears to be true for both seizure efficacy as well as for side effect profile. Nonetheless, two AEDs with virtually identical mechanisms of actions, such as two barbiturates, perhaps should be avoided to minimize side effects.

## 4. Animal Data

Animal studies have demonstrated that certain combinations of AEDs are synergistic for efficacy, while potentially showing antagonism regarding adverse side effects. One rational method of studying synergism is the isobolographic method. The ED_50_ (the dose at which 50% of mice are protected against seizures) of a candidate drug A is calculated. Then a second drug B is added in fixed combinations (typically 3:1, 1:1, and 1:3); ED_50_ for drug B is also calculated. The ED_50_ of the drug A at each combination would decrease linearly if the two drugs are purely additive in effect. If there is synergy, the ED_50_ of the combination would be less than predicted; if there is antagonism, the ED_50_ would be greater. Variations of these models with corrections for nonlinear additivity have been introduced [25,26]. Similarly, side effect profiles can be calculated as the TD_50_, the dose at which a 50% of mice exhibit neurotoxic effect. In this case, antagonism, resulting in a greater than expected TD_50_, would indicate a favorable interaction. Typically, side effects are tested by the Chimney Test, in which motor impairment is measured by the mouse’s inability to climb backwards up a tube. These studies may provide preclinical clues for those combinations that may be effective, and at which doses. These methods have been utilized in the 6 Hz psychomotor seizure model [25], pentylenetetrazole-induced [27], and maximal electroshock-induced seizures [28]. 

Some combinations are synergistic for certain mouse models but not for others (ex. LEV and PB is synergistic for the 6 Hz model [25] but not for the MES model [29]), underscoring the fact that none of the animal models are perfect analogues to human epilepsy or for side effect assessments. In some of these studies, side effects are not as fully evaluated as the antiepileptic properties of drug combinations. Some AEDs were tested more systematically than others, which may explain why they appear overrepresented in the list (See Table 2). In addition, these studies will not uncover combinations for which one AED may alleviate an idiosyncratic side effect of another (e.g., LTG ameliorating mood disturbances of LEV).

Given these limitations, the isobolographic method appears to have identified virtually all known postulated synergistic and antagonistic AED combinations from the limited number of human studies and anecdotal reports. As such, particularly promising combinations not typically used clinically due to perceived intolerability (LTG and topiramate (TPM) for example) should be examined further in human studies.

**Table 2:** AED combinations determined by isobolographic studies in animals.

Favorable AED combinations	
CBZ	GBP [30]*&
	LEV [29]
	OXC [29]
	TPM [31]
	VPA [32]
CZP	OXC [33]
ESX	VPA [27,34]*
FBM	LEV [35]
	LTG [36]
	TPM [37]^&
GBP	LEV [38]
	LTG [30]*
	OXC [39]^&
	PB [30]
	PHT [30]
	TGB [40]
	TPM [41]
	VIG [42]^
	VPA [30]^&
LEV	CBZ [29]
	FBM [35]
	OXC [29]^
	PB [25]
	TPM [29]^
LTG	FBM [36]
	GBP [30]*
	TPM [43]*^&
	VPA [43]*^&
OXC	CBZ [29]
	CZP [33]
	GBP [39]^&
	LEV [29]^
	TPM [37]
PB	GBP [30]
	LEV [25]
	PHT [44]*
PHT	GBP [30]
	PB [44]*
	VPA [31]
TGB	GBP [40]
	VIG [45]*
	VPA [46]
TPM	CBZ [31]
	FBM^&
	GBP [41]
	LEV [29]^
	LTG [43]*^&
	OXC [37]
	VPA [31]
VIG	GBP [42]^
	TGB [45]*
VPA	CBZ [32]
	ESX [27,34]*
	GBP [30]^&
	LTG [43]*^&
	PHT [31]
	TGB [46]
	TPM [31]
**Unfavorable AED combinations**	
CBZ	LTG [43]
CZP	FBM [47]
FBM	CBZ [47]
	OXC [37]!
	TGB [39]
	VPA [47]
LTG	CBZ [43]*!
	OXC [37]*!
OXC	FBM [37]!
	LTG [37]
	PHT [33]!
PHT	OXC [33]!
TGB	FBM [39]
VPA	FBM [47]

*: agreement with available human studies or case reports; ^: particularly favorable combinations; &: both synergy against seizures and antagonism for side effects (the most favorable);!: antagonism against seizures and synergy for side effects (the most unfavorable)

## 5. Human Data

Two polytherapy human trials investigating AEDs are particularly noteworthy. The first attempted to determine whether polytherapy was superior to monotherapy in new onset seizures. Deckers *et al*. [48] conducted a double blind placebo controlled clinical trial of 130 adult-onset epilepsy patients comparing CBZ monotherapy to a combination of CBZ/VPA. The authors hypothesized that the combination of low doses of CBZ and VPA would have fewer side effects than CBZ monotherapy. No statistical difference between the two treatments was found for seizure frequency or drug toxicity. There was a nonsignificant trend of fewer patients on polytherapy withdrawing from the study because of adverse side effects. This trial is remarkable in demonstrating that with equal drug loads, polytherapy can be as effective, and not necessarily more toxic than monotherapy [49]. That this study did not show superior efficacy is not surprising given that most new onset epilepsy patients achieved control of their seizures as expected in this study. The design of this study did not allow for true pharmacodynamic synergy to emerge, as this would have required a lower total drug load for those patients on polytherapy. In addition, the concept of drug load remains poorly clarified [21].

The second trial attempted to determine whether polytherapy was superior to monotherapy in patients who failed monotherapy. Beghi *et al*. [50], using a pragmatic trial designed to mimic clinical practice, randomized 157 patients with uncontrolled seizures on monotherapy to alternative monotherapy *versus* adding an adjunctive AED to the existing treatment. The choice of the AED, whether substituted or added, was left to the treating physician. No significant difference in rates of seizure freedom, retention, or adverse events was found between groups, although trends towards greater seizure freedom (16% *vs.* 14%) and retention (65% *vs.* 55%) at 12 months were seen with polytherapy. Additionally, there was no difference in rate of adverse events. The study was terminated because an interim analysis revealed that effect size of the primary endpoint (retention) was smaller than anticipated, and in combination with poor funding, statistically significant endpoints were not thought to be achievable. Limitations of these two studies were that they mostly included older AEDs, and due to the pragmatic nature of study design, CBZ/LTG was the second most common combination, despite the suspected antagonistic effects. Similar results were seen in a smaller study [51].

Few other systematic studies are available examining comparative effectiveness of polytherapy in humans. A retrospective review of 1,617 seizure free patients revealed that 21% were on polytherapy [52]. Of these, the most common combinations were LTG/VPA, PB/PHT, CBZ/GBP, and CBZ/VPA. However, these pairs may have been the most frequently attempted combinations, or milder, relatively easy to control patients who responded to the first combination of AEDs. Of note, seizure freedom was achieved with CBZ/LTG polytherapy in 14% of these patients, a combination that is believed to be potentially undesirable because of excessive side effects in both human and animal studies [43,53,54,55,56]. A study of 193 patients with focal epilepsy on combination therapy at a single institution in Finland claimed seizure freedom in 37 of 135 patients (27%) on dual AED therapy (specifically five of 19 were on LTG/VPA and three of five were on LEV/LTG). For those on three AEDs, five of 50 patients (10%) were seizure free. A survey of AED use in one referral center in Norway again found LTG/VPA, LEV/LTG, LEV/VPA, and TPM/VPA to be most frequent used [57]. A retrospective study published in abstract form examined 379 patients receiving 2-drug polytherapy who achieved seizure freedom. Favorable combinations were believed to include of PHT/PB and CBZ/VPA. PHT/TPM was found to be potentially ineffective [58]. 

There are a handful of studies directly demonstrating synergistic mechanisms of polytherapy with specific AED combinations. In an open label trial, 347 patients with epilepsy intractable to VPA, CBZ, PHT, or PB monotherapy had LTG added in combination [59]. LTG when added to VPA resulted in significantly greater response (50% or more seizure reduction) than for CBZ or PHT. Although the authors postulate that increased LTG concentration due to the clearance inhibition effects of VPA may have contributed to the greater efficacy, subsequent studies have demonstrated that the results are not purely due to the pharmacokinetic interaction. In a prospective study in which VPA was added to the baseline medication in patients with refractory epilepsy, then substituted with LTG if not responsive, and VPA was finally added back, four of 13 patients who did not respond to either VPA or LTG additions responded to the combination of the two despite serum levels of each drug documented lower than either drug individually [60]. Other studies have suggested that less peak-to-trough fluctuations may contribute to the superior efficacy of the VPA/LTG combination [61]. Similar benefits of this combination have been confirmed in several additional smaller studies [62,63].

With the dearth of definitive large scale studies, and given the large number of possible permutations of combinations, case series may provide some insight into useful combinations. Synergy between VPA and ethosuximide (ESX) in patients with refractory absence seizures has been demonstrated in one study [64]. In an older prospective double-blind study, combination of PHT and PB was found to be effective [13,65]. Smaller series report the following combinations as useful: CBZ/VPA [66]; vigabatrin (VIG)/LTG [67], tiagabin (TGB)/VIG [17], GBP/LTG, and LTG/TPM [68].

The best studied antagonistic combination is LTG and CBZ. Although it was initially proposed to be a pharmacokinetic effect due to the increase in the toxic epoxy residue of CBZ as a result of LTG administration [56], further studies have not found this to be the case, and this unfavorable combination is more likely a pharmacodynamic effect [53,54,55].

A recent cross-sectional study examined the difference in adverse effects in patients on monotherapy *versus* polytherapy [69]. No differences in adverse effects as reported spontaneously or through questionnaire were found between monotherapy and polytherapy. Although patients on polytherapy had higher drug burden, there was no correlation between adverse events and total drug dosage, possibly reflecting the skill of the neurologist in obtaining maximal efficacy with minimal side effects in a non-trial setting.

The studies above highlight the difficulties in rational polytherapy for uncontrolled epilepsy. They have not provided evidence that polytherapy is more efficacious than monotherapy. However, therapeutic dosing of AED polytherapy does not necessarily cause more side effects than monotherapy when performed judiciously. As such, careful selection of favorable drug combinations to optimize efficacy while minimizing side effects is essential.

## 6. Clinical Considerations with Illustrative Case Scenarios

While rational polytherapy remains an elusive goal, guidelines for the approach to monotherapy should also apply for combinations of AEDs, although there would potentially be exaggerated clinical pitfalls: 

(a) It is important for those patients on AED polytherapy as for monotherapy for neurologists to classify the epilepsy carefully, especially if a syndrome can be identified, to implement the most optimal AED management. Although broad spectrum AEDs are easiest to use in patients with multiple seizure types, narrow spectrum AEDs may be added for additional benefit. Adding ESX for absence seizures in patients with an idiopathic generalized epilepsy who may require other AEDs for control of convulsive seizures, is a common scenario. In a patient with Dravet syndrome, polytherapy with topiramate may be particularly helpful whereas carbamazepine or lamotrigine should be avoided [70].

(b) Even with polytherapy, the lowest effective doses of AEDs should be attempted. Once the decision for polytherapy has been made for patients who have failed high dose monotherapy, when the second agent has reached a low therapeutic level, lowering the dosage of the initial agent is advisable. Seizure control may be achieved with plasma levels lower than those usually recommended in the setting of polytherapy, as suggested by the potentially lower levels necessary for effectiveness in add-on therapy *versus* monotherapy for some AEDs [71].

(c) Pharmacokinetic interactions that influence absorption, distribution, or elimination of the affected drug should be carefully evaluated. This is especially true for AEDs affecting hepatic drug metabolizing enzymes [72], and for the elderly who are more likely on multiple medications, and for whom pharmacokinetics may be unpredictable. Plasma AED monitoring becomes of much greater importance, especially when one of the AEDs exerts an enzymatic change or both drugs are protein bound. Therefore, measurement of plasma levels before and after instituting polytherapy is of importance. 

(d) Concurrent medical conditions such as depression or migraine should be considered carefully. Polytherapy should be avoided if possible during pregnancy [73] especially if it includes VPA [74]. 

(e) For patients who fail low dose monotherapy, there are as yet, no data to determine whether high dose monotherapy, low dose alternate monotherapy, or low dose polytherapy leads to best seizure control or lowest side effects. Although AEDs that are used in trials of adjunctive therapy show dose escalation response to both seizure efficacy as well as side effect proliferation, most patients responding to an AED will do so at a low dose [75,76]. Further studies are needed here. In the absence of side effects at a low dose, it is reasonable to increase the dose of monotherapy into a higher range especially if seizures partially responded. However, if there is concern that high dose monotherapy would cause excessive side effects or there has been no impact on seizures, an alternate low dose monotherapy should be explored. Low dose polytherapy should be considered after failure of two monotherapy trials because of either lack of efficacy or side effects at higher doses. It may be considered earlier if low dose of the first monotherapy results in substantial improvement in seizures but a higher dose can not be tolerated. Patients should be counseled that if carefully prescribed, there is little evidence that the greater number of AEDs automatically confers greater side effect risk. 

(f) Polytherapy will increase potential pitfalls associated with monotherapy [77]. The patient may be less compliant with a more complex regimen. Medication cost will invariably be greater. Care should be taken to minimize medication errors. These factors should be taken into account prior to administering polytherapy, especially in patients who may have limited cognition and limited resources.

We present several illustrative case scenarios demonstrating possible useful approaches to polytherapy:

*Case scenario*

*A 24 year old man with lifelong partial epilepsy had daily seizures when he was first evaluated at our institution. His AED regimen consisted of high dose VPA, LEV, and ESX. A combination of VPA and LTG was started and both LEV and ESX were removed, with marked improvement, though not complete control, of his seizures. Serum VPA level remained similar throughout the course of his evaluation.*

There are few specific medications shown to have synergistic pharmacodynamic interaction; the definite favorable combination of LTG and VPA is the exception. Although the combination of CBZ and LTG has been demonstrated to have less than ideal interaction, studies have shown this to be a combination with considerable success perhaps because of sheer numbers of patients placed on them [52]. As such, although it may be reasonable to attempt to try to achieve polytherapy with combinations of known pharmacodynamic advantages and to avoid combinations that have been suspected of being antagonistic, these guidelines should not be strictly followed until more data are forthcoming.

*Case scenario*

*A 56-year-old man S/P resection of an arteriovenous malformation with continued seizures was started on LEV during hospitalization. He experienced profound neuropsychiatric side effects and was prescribed LTG with the intent to convert him to monotherapy. The neuropsychiatric symptoms resolved with low dose of LTG (100 mg a day). Upon reaching a therapeutic dose of LTG (150 mg twice a day), LEV was tapered off. One week later, he experienced a recurrent seizure. Upon his request, LEV was restarted without change in LTG. His neuropsychiatric side effects never recurred and he has been seizure and side effect free on LEV/LTG.*

At times, one may be able to negate the side effect of one AED with the addition of another. This may be particularly true with idiosyncratic side effects. Although there are no known antiepileptic synergistic effects of the combination of LEV/LTG, the common and disturbing neuropsychiatric side effects of LEV can sometimes ameliorate with the addition of LTG likely due to LTG’s mood stabilizing properties (anecdotal observation). Similarly, we have observed that TPM can partially counteract the weight gain encountered by the use of VPA.

*Case scenario*

*A 29 year old man with lifelong refractory epilepsy with daily seizures on PHT, pregabalin (PGB), OXC, and TGB was started on lacosamide (LAC) while PGB was tapered off. He experienced a marked reduction of seizures, although complained of severe dizziness. His dizziness resolved after moving his regimen from 8 am and 10 pm to strictly 12 hours apart, and by separating OXC and LAC intake by 2 hours.*

Despite the urgency to achieve seizure control, the importance of careful side effect assessment cannot be overemphasized. Side effects are potentially amenable to changing dose scheduling regimen which is a far simpler enterprise. If desired seizure control is achieved, side effects may be modulated by stricter adherence to q12h dosing, staggering component AED intake by several hours, by shifting to a greater evening dosage, or by using extended release formulations [78,79], without potentially decreasing seizure control efficacy.

*Case scenario*

*A 30 year old woman with absence epilepsy from childhood discontinued VPA despite efficacy because of weight gain. She remained on a regimen of clonazepam (CZP), TPM and zonisamide (ZNS). A trial of ESX 250 mg bid resulted in severe side effects and no improvement in seizures. TPM was tapered off for a planned pregnancy. While transiently improved during pregnancy, seizures became more frequent after delivery. A second attempt with ESX was tried and this time she became seizure free, without significant side effects.*

One important question is whether any polytherapy with three or more AEDs can ever be rational. Three drug regimen are generally avoided if possible [80]. Indeed, the vast majority of patients reaching seizure freedom do so with two AEDs, and virtually no one achieves seizure freedom with four AEDs [52]. If a patient is on four or more AEDs, a concerted attempt should be made to reduce the regimen to two or three AEDs, possibly in an inpatient hospital setting. It is difficult to convince patients or their caretakers that removing a drug would lead to lowering the dose of the other drugs with improved efficacy (e.g., removal of PHT in a patient taking LTG). Unfortunately, it is often difficult to determine which medication to eliminate when a patient has been on chronic polytherapy. In such instances, simplification of regimen may need to be performed in the epilepsy monitoring unit. A transient increase in seizures may occur, and this may need to be tolerated before withdrawal can be deemed unsuccessful.

*Case scenario*

*A 22 year old man with refractory partial epilepsy and unremarkable MRI brain underwent left temporal lobectomy. Complex partial seizures remitted, but prolonged convulsions began to occur without warning every two weeks, occasionally leading to status epilepticus and hypoxia, which was worse than his condition prior to surgery. Multiple medications in combination were tried and were ineffective, with the longest seizure freedom being two months. He was then advised to rotate his medications by maintaining LEV and CZP, and rapidly titrating either GBP, LTG, or ZNS for 2.5 months at a time before removing. He has been completely seizure free for over three years.*

Many patients experience transient but excellent control of seizures after the introduction of an AED for months before seizures recur, and the concept of intermittent administration of AEDs has been considered, and in several instances, successfully implemented [81,82]. Our case demonstrates successful rapid cycling with two AEDs. This potentially difficult to implement strategy may be performed with AEDs that can be administered rapidly at near-therapeutic doses.

*Case scenario*

*A 50-year-old man experienced a complex partial seizure with secondary generalization. Subsequent evaluation revealed a right parietal/temporal glioblastoma. After surgery, he continued to experience frequent sensory seizures consisting of waves of numbness down his left side. He was placed on high dose of LEV, VPA, and TPM without resolution of these events but with increasing drowsiness and dizziness. He was tapered down to low dose LEV on which he continued to have more frequent simple partial seizures but much improved cognition. He had no further complex partial or generalized seizures until shortly prior to his death 14 months later.*

Highly intractable seizures present both a practical and intellectual dilemma. The goal of eradication of all simple partial seizures for patients with lesional epilepsy (often from brain tumors) often leads to excessive AEDs and accumulation of side effects from polytherapy. Similarly, this may also need to be tempered in patients with severe symptomatic generalized epilepsies and underlying cognitive difficulties. Reasonable expectations need to be set for both the patient as well as the treating physician, and it may be preferable to administer AEDs to control only the most severe seizures.

## 7. Conclusions

Polytherapy remains the reality for a large proportion of patients with epilepsy. Rational polytherapy is still a difficult, elusive goal in the management of these patients. More research is needed in this area; investigations of mechanism of action of AEDs and animal studies have not yet clearly delineated the core principles governing effective polytherapy in humans; in the future, pharmacogenomics, may provide further guidance as to which combinations may potentially be efficacious. Identification of favorable combinations, particularly those involving idiosyncratic unpredicted effects, may still result largely from clinical observations. In the meantime, successful polytherapy requires treatment to be tailored on an individual basis.
